# Characterization of *PDAT* Genes in Oat (*Avena sativa* L.) and the Role of *AsPDAT-5C* in Lipid Biosynthesis and Abiotic Stress Response

**DOI:** 10.3390/plants15010035

**Published:** 2025-12-22

**Authors:** Yan Sun, Jinzhou Yang, Ruirui Hu, Chen Li, Qian Yang, Xiping Sun, Zhiwei Zhang, Runzhi Li, Jinai Xue

**Affiliations:** 1 College of Agriculture, Shanxi Engineering Research Center for Genetics and Metabolism of Specific Crops, Shanxi Agricultural University, Taigu 030801, China; 232006@sxau.edu.cn (Y.S.); 15758210505@163.com (J.Y.); hrr20230902@126.com (R.H.); 13720953629@163.com (C.L.); 13934105234@163.com (Q.Y.); sxpljj@126.com (X.S.); rli2001@126.com (R.L.); 2 College of Forestry, Shanxi Agricultural University, Taigu 030801, China

**Keywords:** *Avena sativa*, phospholipid:diacylglycerol acyltransferase, lipid accumulation, stress response

## Abstract

Phospholipid:Diacylglycerol Acyltransferase (PDAT) catalyzes the final step of the acyl-CoA-independent triacylglycerol (TAG) biosynthesis pathway and plays an important role in lipid metabolism and abiotic stress responses in plants. Oat (*Avena sativa* L.) possesses the highest lipid content among cereal crops, yet the functions of *PDAT* genes in this species remain largely unexplored. In this study, we identified and characterized three *AsPDAT* genes in oat, which form a homeologous triplet evenly distributed across the three subgenomes and show high conservation in sequence and gene structure. Phylogenetic analysis indicated a clear divergence between monocot and dicot PDATs. Expression profiling revealed that the three *AsPDAT* genes share similar organ-specific and stress-responsive expression patterns, suggesting functional conservation following polyploidization, with *AsPDAT-5C* showing relatively higher transcript levels. The enzymatic activity of AsPDAT-5C was confirmed by complementation of the TAG-deficient yeast quadruple mutant H1246. Transient expression in *Nicotiana benthamiana* epidermal cells demonstrated that AsPDAT-5C localizes to the endoplasmic reticulum. Stable overexpression of *AsPDAT-5C* in *Nicotiana tabacum* significantly increased lipid content in both leaves and seeds without compromising plant growth and enhanced tolerance to cold and phosphorus-deficiency stresses. Our results provide new insights into the AsPDAT gene family and underscore the potential of AsPDAT-5C in engineering lipid biosynthesis and improving stress resilience in plants.

## 1. Introduction

Plant lipids are an essential nutritional resource for humans and animals and a vital renewable feedstock for biodiesel and industrial applications. [1]. Triacylglycerol (TAG), the predominant storage lipid in mature seeds, is the final metabolic product of fatty acid biosynthesis [1]. The last committed step of TAG assembly is primarily catalyzed by two classes of acyltransferases: Diacylglycerol Acyltransferase (DGAT) and Phospholipid:Diacylglycerol Acyltransferase (PDAT), which utilize acyl-CoA and phospholipids as acyl donors, respectively [2].

PDATs are structurally conserved and share high sequence similarity with mammalian lecithin:cholesterol acyltransferase (LCAT) family members [3]. As previous studies have shown, *PDAT* genes often exist as multiple copies in plant genomes [4]. The first *PDAT* gene was cloned from yeast based on sequence homology to human *LCAT* [5], and *PDAT* was subsequently identified in the model plant Arabidopsis [6,7] as well as in several oilseed crops and oleaginous microalgae [8,9,10]. So far, genetic modification of *PDAT* genes has been considered a promising strategy for engineering lipid production. Overexpression and knockout of yeast *PDAT* (*LRO1*) supported its indispensable contribution to lipid accumulation at the exponential growth stage, while *DGAT2* (*DGA1*) contributed predominantly during the stationary growth phase [11]. In contrast to the situation in yeast, however, AtPDAT1 activity encoded by At5g13640 was not a major determining factor for seed lipid accumulation, but had overlapping function with AtDGAT1 in normal pollen and seed development in Arabidopsis [12]. Notably, *AtPDAT1* was highly expressed not only in seeds but also in vegetative organs, and it has been shown to be crucial for lipid biosynthesis in fast-growing plant organs [13]. Furthermore, heterologous overexpression of *SsPDAT1* from *Sapium sebiferum* increased total lipid content in *Brassica napus* seeds [14], while overexpression of an endogenous *PDAT* gene in *B. napus* resulted in a more modest increase of 1.86–2.77% [15]. In *Xanthoceras sorbifolium*, expression analyses showed that the *XsPDAT1* gene was induced under conditions that positively correlated with lipid accumulation, implicating its involvement in regulating lipid biosynthesis [16]. Although similar responses of *PDAT* genes in boosting lipid synthesis have also been reported in microalgae, they function primarily under unfavorable environmental conditions [10,17].

Recent studies have also proposed the involvement of *PDAT* genes in plant tolerance to various abiotic stresses [3]. For example, the overexpression of *AtPDAT1* led to substantially increased thermotolerance, and this response was notably compromised in *atpdat1* mutants [18]. A recent study further indicated that AtPDAT1 was critical for maintaining membrane homeostasis under cold stress by remodeling lipid metabolism [19]. Beyond temperature stress, PDATs were also thought to be key regulators in response to other environmental perturbations, such as salt, drought, light, and wounding, as suggested by stress-inducible expression patterns of *CsPDATs* in *Camelina sativa* and *OepPDATs* in olive and the association with lipid metabolism [20,21]. Nevertheless, *PDAT* genes remain functionally characterized in only a limited number of species, and the mechanisms underlying PDAT-mediated stress tolerance are still poorly understood.

Oat (*Avena sativa* L., 2n = 6x = 42, AACCDD), belonging to the genus *Avena* within the grass family Poaceae, is the seventh most important cereal crop in terms of worldwide production, and widely used as an energy and nutrient source for human food and animal feed [22]. It exhibits strong environmental adaptability and accumulates abundant metabolites, including soluble dietary fiber, polyunsaturated fatty acids, vitamins, alkaloids, polyphenols and minerals, which determine the nutritional and health benefits of oat [22]. Particularly, oat seed lipid content is the highest (up to 18%) within the cereal species, and the accumulated lipid is predominantly (up to 90%) confined to the starchy endosperm, which has attracted the attention of researchers for the development of high-oil oat varieties as a sustainable oil production [23,24]. Also, several gene expression studies of developing oat seeds have been conducted, which have led to the identification of a number of promising targets for lipid improvement in oat [25,26]. However, to date, genes contributing to lipid accumulation in oat endosperm have not been functionally characterized.

In this study, we systematically investigated the PDAT family in oat, which has not been previously described. The oat genome contained three homologous copies of *AsPDAT* genes caused by polyploidization, which tend to possess functional redundancy because of sequence similarity relationships and similar expression patterns. Overexpression of *AsPDAT-5C* in tobacco significantly increased lipid levels in both seed and leaf organs. We further characterized the role of *AsPDAT-5C* in regulating abiotic stress resistance, including cold and phosphorus deficiency. Collectively, the identification of *AsPDAT-5C* provides a promising genetic resource for enhancing lipid yield. Our work further reveals the role of *AsPDAT-5C* in plant adaptation to abiotic stresses by demonstrating its impact on lipid metabolism.

## 2. Results

### 2.1. Genome-Wide Identification and Sequence Analysis of PDATs in Oat

To identify PDAT members in oat, the protein sequences Arabidopsis PDAT (AtPDAT1/At5g13640 and AtPDAT2/At3g44830) were used as queries for BLAST search against the oat genome. It is well known that cultivated oat is a hexaploid species with most genes present as triplicate homoeologs, which are derived from hybridization between its diploid and tetraploid progenitor species. Accordingly, we identified three putative PDAT members forming a collinear homeologous set located on chromosomes 5A, 5C, and 5D, which were designated AsPDAT-5A, AsPDAT-5C, and AsPDAT-5D based on their chromosomal origins. The deduced amino acid sequences of the three AsPDATs shared high sequence similarity (96–98%). EMBOSS Needle pairwise alignment showed that AsPDAT-5A, AsPDAT-5C, and AsPDAT-5D share 73.2%, 77.2%, and 76.9% amino acid identity with AtPDAT1, respectively, but less than 60% identity with AtPDAT2. We also computed basic physicochemical properties for each protein, including amino acid number, molecular weight (MW), theoretical isoelectric point (pI), and aliphatic index (Table 1). Subcellular localization predictions indicated that all three AsPDATs are likely endoplasmic reticulum (ER)-localized proteins.

**Figure 1:**
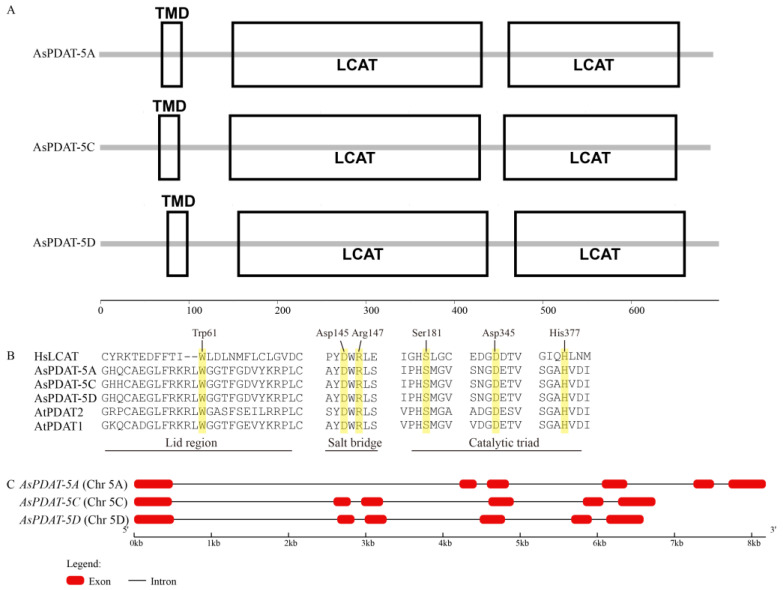
Bioinformatics analyses of AsPDAT sequences. (**A**): Schematic representation of predicted AsPDAT protein structures. TMD, Transmembrane domain; LCAT, Lecithin-Cholesterol Acyltransferase domain. (**B**): Multiple sequence alignment of oat AsPDATs and Arabidopsis AtPDATs with human lecithin:cholesterol acyltransferase (HsLCAT). Structurally conserved LCAT motifs, including the Lid region (Trp-61), a Salt Bridge between Asp-145 and Arg-147, and a Catalytic Triad (Ser-181-Asp-345-His-377), are indicated above the alignment. Conserved residues are highlighted in yellow. (**C**): Exon–intron organization of *AsPDAT* genes in oat.

All three AsPDAT homoeologs were predicted to contain an N-terminal transmembrane domain (TMD) composed of 21 amino-acid residues and a conserved lecithin:cholesterol acyltransferase (LCAT) domain (Figure 1A). The LCAT domain is characteristic of lipid-metabolizing enzymes such as mammalian LCAT and plant PDAT. Multiple sequence alignment of oat AsPDATs, Arabidopsis AtPDATs, and human HsLCAT showed that PDATs shared the functionally important features of LCAT enzyme, including Trp-61 within the ‘lid’ region, a salt bridge and the catalytic Ser-Asp-His triad (Figure 1B). It is worth mentioning that the residues surrounding these conserved sites diverge considerably between plant PDATs and human LCAT (Figure 1B), which may contribute to the formation of novel structures and functions of plant PDAT enzymes during evolution. Based on the well-assembled reference genome, we found that all three *AsPDAT* genes had similar exon-intron organization, each consisting of six exons and five introns (Figure 1C).

### 2.2. Phylogenetic Analysis of PDATs in Oat

**Figure 2:**
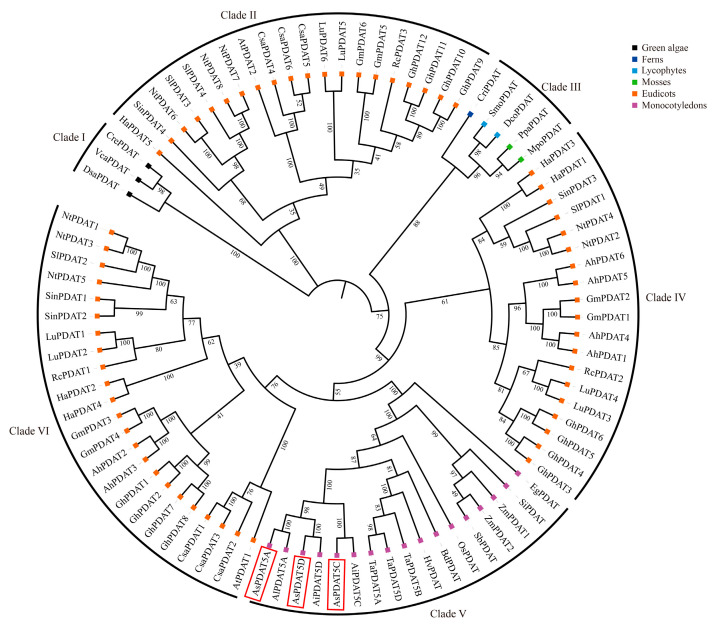
Maximum Likelihood (ML)-based phylogenetic tree of PDAT proteins from representative plant and algal species. PDATs are grouped into six major clades (I–VI). Colorful squares at the tips represent different plant taxa. Bootstrap support values are given as a percentage of 1000 replicates at the branch nodes. Species and data information for PDAT sequences used in this study are presented in Supplementary Table S1. The three oat *AsPDAT* members are highlighted with a red box.

A phylogenetic tree constructed from representative eudicots, monocots, mosses, ferns, lycophytes, and green algae showed that PDATs could be separated into six distinct clades, each of which was further subdivided into two or more subclades (Figure 2). Algal PDATs were phylogenetically separated from land plant PDATs and grouped at the basal position of the tree. Clade III included PDAT orthologs from mosses, ferns and lycophytes, whereas Clade V was exclusive to monocots. Eudicot PDATs did not cluster together and were distributed across three different clades: clades II, IV, and VI, with each clade consisting of members from a mixture of species. Interestingly, clade VI within eudicots was phylogenetically closer to the monocot clade than to other eudicot PDATs. It was further found that PDAT copy numbers varied widely across different plant species. In summary, green algae, mosses, ferns, and lycophytes generally contained a single PDAT in each species. Within monocots, most (sub)genomes also possessed one single *PDAT* gene, with the only exception being maize, which is a diploid species but contains two *PDAT* copies. In contrast, *PDAT* genes have expanded notably in eudicots. As a hexaploid plant, the oat genome contained three *AsPDAT* homeologs, corresponding to the sum found in its ancestors, namely, one in diploid *Avena longiglumis* and two in tetraploid *Avena insularis*. The phylogenetic reconstruction indicated that oat *AsPDAT* genes originated from its ancestral lineages (Figure 2).

### 2.3. Expression Analysis of AsPDAT Genes in Oat

**Figure 3:**
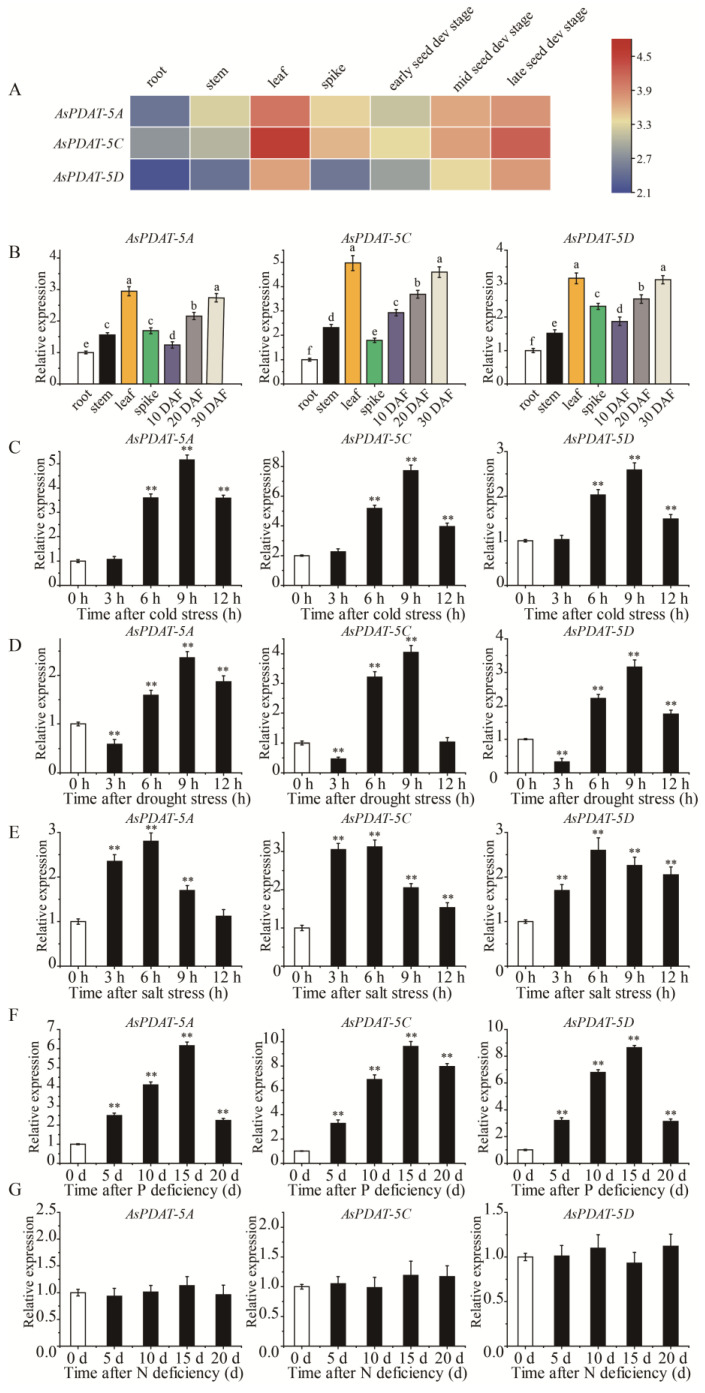
Expression profiles of oat *PDAT* genes. (**A**): Heatmap showing the expression profiles of *AsPDAT genes* in different organs and developmental stages based on Log_2_ FPKM values in the transcriptome data. Blue and pink indicate low and high transcript abundance, respectively. (**B**): Relative expression levels of *AsPDAT-5A*, *AsPDAT-5C*, and *AsPDAT-5D* in different organs, including developing seeds, measured by qRT-PCR. *AsActin* gene was used as an internal reference. The relative expression level for each gene was calculated with the 2^−ΔΔCT^ method. Each value represented the mean of 3 replicates, and expression was normalized to the root sample (set to 1). Different alphabetical letters represent significant differences at *p* < 0.05 (one-way ANOVA). (**C**–**G**): Expression of *AsPDAT* genes in oat leaves under stress treatments at different time points, including cold (**C**), drought (**D**), salt (**E**), P deficiency (**F**), and N deficiency (**G**), based on qRT-PCR. *AsActin* gene was used as an internal reference. The relative expression level for each gene was calculated with the 2^−ΔΔCT^ method. Each value represented the mean of 3 replicates. Significant differences compared with 0 h control were determined using Student’s *t*-test. The asterisk indicates statistically significant differences: ** *p* < 0.01.

As the first step to explore the potential functions of *AsPDAT* genes in oat, we examined their expression across different organs and under diverse environmental conditions. We firstly utilized the publicly available transcriptome data from oat to detect the expression patterns of *AsPDAT* genes in root, stem, leaf, spike, and seeds at three developmental stages. Fragments per kilobase of transcript per million mapped reads (FPKM) values showed that all three *AsPDAT* homeologs shared similar spatial expression patterns, with detectable transcripts in all organs and comparatively higher expression in leaves and during late seed development (Figure 3A). Among them, *AsPDAT-5C* was more highly expressed than the other two *AsPDAT* genes (Figure 3A). Additionally, we performed quantitative real-time PCR (qRT-PCR) to validate these patterns. Consistent with the transcriptome data, all three *AsPDAT* genes were preferentially expressed in leaves and developing seeds (Figure 3B). Interestingly, each gene displayed coordinated transcriptional changes under several abiotic stresses (Figure 3C–G). Under cold stress, the expression levels of *AsPDAT* genes were basically unaffected during the first 3 h, then sharply increased with the peak at 9 h, and then declined (Figure 3C). We also confirmed that *AsPDAT* genes were transcriptionally induced at 5 d after phosphorus (P) deficiency, with maximal levels at 15 days followed by a decrease (Figure 3F). In addition, *AsPDAT* genes were significantly altered under drought (Figure 3D) and saline (Figure 3E) conditions, but not under nitrogen (N) deficiency (Figure 3G). Collectively, these findings indicate that the three *AsPDAT* genes may function similarly in response to multiple stresses. Notably, *AsPDAT-5C* was the most strongly induced under both cold and P-deficient conditions (Figure 3C,F), underscoring its potential role in adaptation to these specific stresses.

### 2.4. AsPDAT-5C Is Localized to Endoplasmic Reticulum and Exhibits Acyltransferase Activity

**Figure 4:**
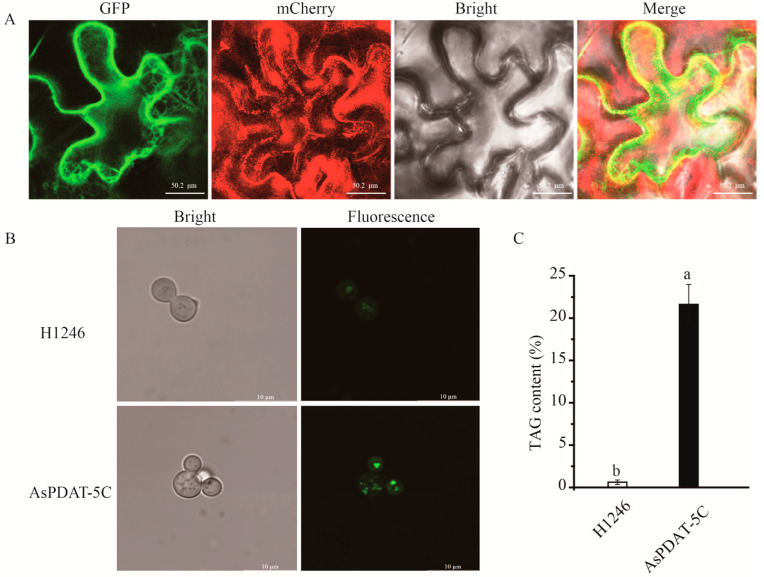
Functional characterization of AsPDAT-5C from oat. (**A**) Subcellular localization of AsPDAT-5C. Confocal microscopy images of *N. benthamiana* epidermal cells co-expressing *AsPDAT-5C*-GFP (green, left panel) and the ER marker mCherry-HDEL (red, second panel), along with the corresponding bright-field (third panel) and merged (right panel) images. The yellow signal in the merged panel confirms co-localization with the ER. (**B**) Neutral lipid accumulation in TAG-deficient *Saccharomyces cerevisiae* mutant H1246 transformed with either the empty vector (pYES2.0) or the pYES2.0-*AsPDAT-5C* construct was induced with galactose and stained with the neutral lipid dye BODIPY 505/515. Fluorescence microscopy reveals lipid droplet formation only in *AsPDAT-5C*-expressing cells. (**C**) Quantitative analysis of TAG content. TAG was extracted from the yeast cells described in (**B**) and quantified. Data are presented as mean ± SD of three independent experiments. Different lowercase letters indicate a statistically significant difference (*p* < 0.05, one-way ANOVA).

To determine the subcellular localization of AsPDAT-5C, the 35S::*AsPDAT-5C*-GFP fusion construct was transiently expressed in *Nicotiana benthamiana* leaf epidermal cells via Agrobacterium-mediated transformation. Confocal microscopy revealed that the green fluorescence signal from the *AsPDAT-5C*-GFP fusion protein extensively overlapped with the signal from the co-expressed endoplasmic reticulum (ER) marker mCherry-HDEL (Figure 4A), confirming the ER localization of AsPDAT-5C.

To investigate whether AsPDAT-5C possesses phospholipid:diacylglycerol acyltransferase (PDAT) activity, its full-length coding sequence was cloned into the yeast expression vector pYES2.0 and expressed in the TAG-deficient *Saccharomyces cerevisiae* quadruple mutant H1246 under the control of a galactose-inducible promoter. Lipid accumulation was visualized using the neutral lipid-specific fluorescent dye BODIPY 505/515. Following galactose induction, H1246 cells expressing *AsPDAT-5C* displayed intense BODIPY fluorescence, indicative of neutral lipid synthesis (Figure 4B). In contrast, control cells transformed with the empty vector showed negligible fluorescence (Figure 4B). Consistent with this phenotypic complementation, quantitative analysis confirmed a significant increase in TAG content in the *AsPDAT-5C*-expressing yeast cells (Figure 4C). Collectively, these results demonstrate that AsPDAT-5C is an ER-localized protein that functions as a PDAT, capable of restoring TAG biosynthesis in the lipid-deficient yeast mutant H1246.

### 2.5. Overexpressing AsPDAT-5C Increased Total Lipid Content in Tobacco (Nicotiana tabacum)

**Figure 5:**
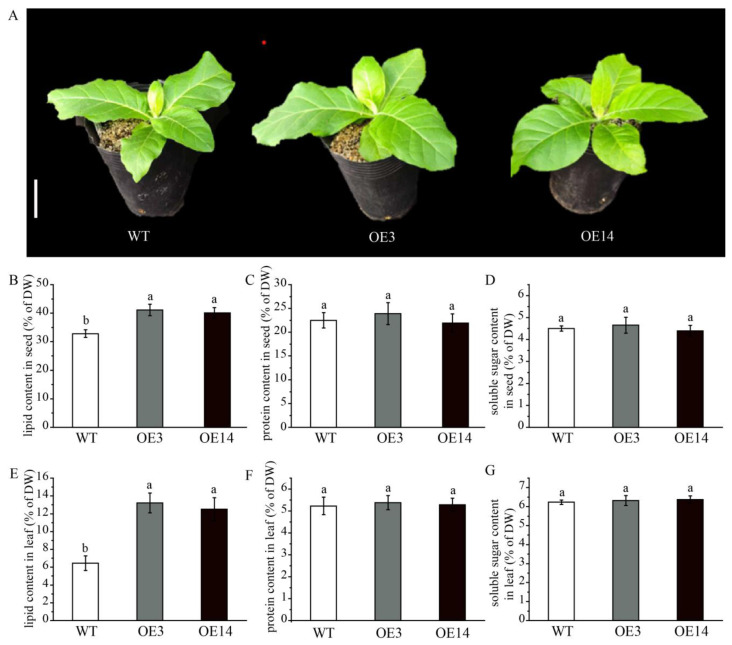
Analysis of the content of lipid, protein and soluble sugar in *AsPDAT-5C*-overexpressing seeds and leaves of *N. tabacum*. (**A**): Phenotypes of wild type (WT) and two independent *AsPDAT-5C*-overexpressing tobacco lines (OE3 and OE14) grown in soil for 28 days after germination. OE3 and OE14 were selected for their significantly higher *AsPDAT-5C* transcript levels (see Supplementary Figure S1). Scale bar indicates 5 cm. (**B**–**D**): Lipid content (**B**), protein content (**C**), and soluble sugar content (**D**) in mature seeds of WT and transgenic tobacco plants. (**E**–**G**): Lipid content (**E**), protein content (**F**), and soluble sugar content (**G**) in mature leaves of WT and transgenic tobacco plants. Data are means ± SD (*n* = 3). ANOVA, complemented by Tukey’s test at a 95% confidence level, was used for data analysis. Distinct lowercase letters above the bars signify significant differences, with a *p*-value of less than 0.05.

To examine the role of *AsPDAT-5C* gene in lipid biosynthesis, we stably introduced a constitutive 35S::*AsPDAT-5C* construct into model plant tobacco (*N. tabacum*) via Agrobacterium-mediated leaf disc assay. From the resulting transgenic lines, two independent lines (OE3 and OE14) showing the highest *AsPDAT-5C* transcript levels were selected for further analysis (Supplementary Figure S1). Under pot-grown conditions, the overexpression lines showed no obvious phenotypic differences from wild-type (WT) plants (Figure 5A). In WT tobacco controls, the total seed lipid content was 32.8% of dry weight (DW), while overexpression of *AsPDAT-5C* led to a significant increase in total seed lipid content to 41.1% in OE3 and 40.08% in OE14 (Figure 5B). A similar increase was also observed in transgenic tobacco leaves, where lipid content rose from 6.46% DW in WT to 13.2% in OE3 and 12.5% in OE14 (Figure 5E). These results demonstrate that *AsPDAT-5C* enhances lipid accumulation in both seed and vegetative organs. In contrast, protein and soluble sugar levels in leaves and seeds of the overexpression lines remained largely unchanged relative to WT (Figure 5C,D,F,G), indicating that the increased lipid content did not come at the expense of these major metabolites.

### 2.6. Overexpression of AsPDAT-5C Enhanced Cold Stress Tolerance

**Figure 6:**
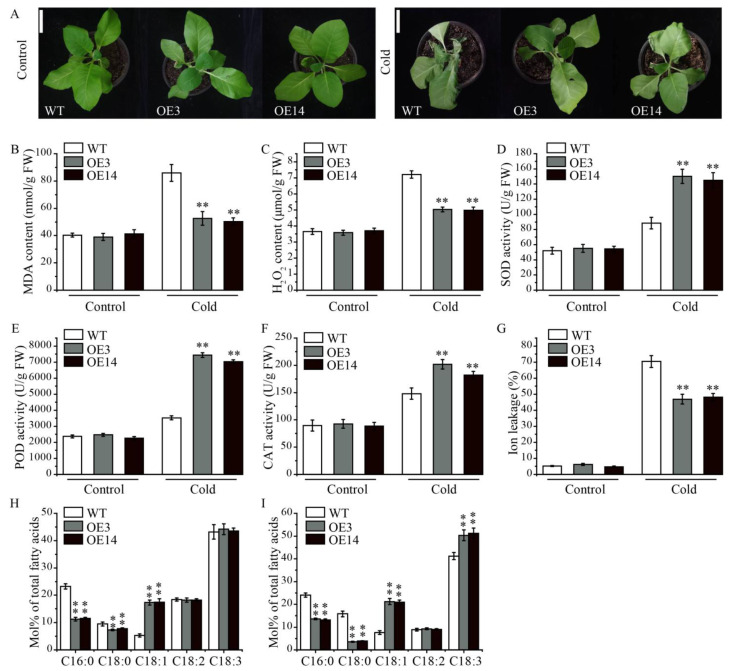
*AsPDAT-5C* overexpression enhanced resilience to cold stress in transgenic tobacco plants. (**A**): Phenotypes of WT and transgenic lines under control and cold conditions. Scale bar indicates 5 cm. (**B**–**G**): MDA content (**B**), H_2_O_2_ content (**C**), SOD activity (**D**), POD activity (**E**), CAT activity (**F**), and ion leakage (**G**) in leaves of WT and *AsPDAT-5C*-OE lines under control and cold stress conditions. (**H**,**I**): Fatty acid composition in leaves of WT and *AsPDAT-5C*-OE lines under control (**H**) and cold stress (**I**) conditions. Data are presented as the molar percentage (mol%) of each fatty acid relative to the total fatty acids. Values are means ± SD (*n* = 3). Significant differences between WT and OE lines under the same condition were determined by Student’s *t*-test (** *p* < 0.01).

Because the expression of *AsPDAT-5C* was stress-inducible, we are interested in exploring whether *AsPDAT-5C* was involved in regulating cold tolerance. Following 3 d of cold exposure, WT seedlings displayed more severe wilting, curling and necrosis compared to the transgenic tobacco lines (Figure 6A). In addition, the MDA content and H_2_O_2_ accumulation were significantly lower in *AsPDAT-5C* overexpressed lines compared to those of the WT (Figure 6B,C), while the enhanced activities of antioxidant enzymes such as superoxide dismutase (SOD), peroxidase (POD), and catalase (CAT) were observed in the seedlings of overexpression plants (Figure 6D–F). Transgenic lines also displayed enhanced cold-tolerance phenotype with reduced ion leakage relative to WT after cold treatment (Figure 6G). To provide a more comprehensive analysis, we conducted measurements on the leaf fatty acid composition. Under normal conditions, overexpression of *AsPDAT-5C* showed a reduction in proportions of C16:0 and C18:0 and an increase in C18:1 (Figure 6H). After cold treatment, transgenic leaves contained significantly less C16:0 and C18:0 and more of the unsaturated fatty acids C18:1 and C18:3 than WT leaves (Figure 6I). These shifts toward a more unsaturated fatty acid profile are consistent with a role for *AsPDAT-5C* in modulating membrane lipid composition to enhance cold tolerance.

### 2.7. Overexpression of AsPDAT-5C Increased Tolerance to P Deficiency

**Figure 7:**
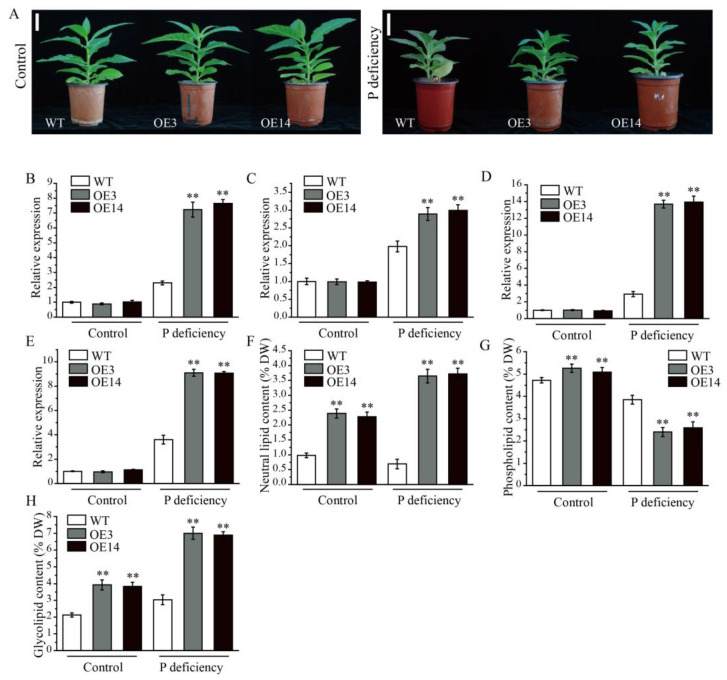
*AsPDAT-5C* overexpression enhanced resilience to P deficiency stress in transgenic tobacco plants. (**A**): Phenotypes of WT and *AsPDAT-5C*-OE plants under control and P-deficient conditions. Scale bar indicates 5 cm. (**B**–**E**): qRT-PCR analysis of *NtPHT1;1* (**B**), *NtPHO1* (**C**), *NtPHR1* (**D**), and *NtMGD2* (**E**) in *AsPDAT-5C* overexpression lines and WT under control and P-deficient conditions. *NtTubulin* (N181029A17) was used as an internal control. Transcript levels of these genes were relative to that in the WT control treatment. The experiment was repeated three times with similar results. Means are presented as bar graphs and error bars represent standard deviation. ** *p* < 0.01. (**F**–**H**): Content of different lipid classes, including neutral lipid (**F**), phospholipid (**G**), and glycolipid (**H**) in leaves of WT and OE lines under control and P-deficient conditions. Data are means ± SD from three biological replicates. Statistical comparisons were performed using Student’s *t*-test (** *p* < 0.01).

To further investigate the stress-related function of *AsPDAT-5C*, the WT and overexpression lines were subjected to P-deficient conditions. After 15 days of P deficiency, transgenic plants exhibited better growth performance, while WT plants showed pronounced growth retardation and leaf shrinkage (Figure 7A). To explore the molecular mechanism underlying this enhanced tolerance, we selected four well-established low P-responsive marker genes, including *NtPHT1;1* (phosphate transporter, GenBank accession number: AF156696), *NtPHO1* (phosphate transporter, GenBank accession number: 107814602), *NtPHR1* (phosphate starvation response regulator, GenBank accession number: 107783857), and *NtMGD2* (monogalactosyldiacylglycerol synthase 2, GenBank accession number: AB047476), and analyzed their expression profiles in leaves of WT and overexpression plants under both control and P-deficient conditions. Under normal P supply, transcript levels of these genes were comparable between WT and overexpression lines. Upon P stress, however, all four genes were significantly induced in both WT and transgenic plants, with their expression consistently and markedly higher in the overexpression lines compared to WT (Figure 7B–E), suggesting that the overexpression of the *AsPDAT-5C* gene, which improved P deficiency tolerance, may be associated with the expression of these stress-responsive marker genes. Consistent with the role of PDAT in lipid metabolism, lipid profiling revealed that under P deficiency, transgenic leaves accumulated less phospholipid but more neutral lipid and galactolipid compared to WT leaves (Figure 7F–H). These results suggest that AsPDAT-5C enhances P deficiency tolerance by remodeling membrane lipid composition, specifically by reducing phospholipid demand and promoting the synthesis of non-phosphorus-containing neutral and galactolipids.

## 3. Discussion

As a widely used feedstock in both food and non-food applications in modern society, enhancing the accumulation of storage lipid (predominantly triacylglycerol, TAG) in plants is of great interest in molecular breeding of oil crops, which has encouraged the identification of gene targets involved in TAG biosynthesis in plants [27]. PDAT is a strong candidate enzyme responsible for the control of TAG biosynthesis in an acyl-CoA-independent manner. In contrast to DGAT, which catalyzes the final step of the acyl-CoA-dependent TAG biosynthesis and has been extensively studied in oilseeds, the biological functions of PDAT remain less clear [3]. Within cereal crops, oat is unique for its unusually high lipid-accumulating capacity in seed, marking its potential as an alternative oil crop worldwide [23]. Motivated by the likely role of PDAT in TAG accumulation, we conducted a comprehensive analysis of the PDAT gene family in oat.

Genome-wide identification revealed a homeologous triplet of *PDAT* genes in oat, and this balanced configuration may have contributed to the stability of TAG metabolism during oat evolution. Taking advantage of publicly available sequenced genomes, we further demonstrated that PDATs are broadly existent in the Viridiplantae (Figure 2), consistent with their indispensable roles in primary metabolism. The topology of the phylogenetic tree allowed us to classify the PDAT proteins into six clades, with most clades having high statistical support (bootstrap > 80%; Figure 2). Within these clades, most internal branches also showed strong support, indicating stable sub-structuring. Interestingly, the large differences in the number of *PDAT* genes in different plant lineages were observed. The eudicot-specific *PDAT* gene expansion indicated no direct link between the number of these genes and their genome size. Phylogenetic analysis revealed that algal PDATs formed a distinct clade from land plants (Figure 2), suggesting that algal PDATs may possess divergent enzymatic functions. This hypothesis was supported by the structural differences between algal and land plant PDATs [10]. For instance, the *Chlamydomonas* PDAT has been reported to exhibit previously unrecognized transacylation and lipase activities using galactolipids and neutral lipids as substrates [10]. Previous studies have also observed the existence of multiple *PDAT* gene copies in different eudicots [4,28], but not all encoded PDATs are functional TAG-synthesizing enzymes, such as AtPDAT2, LuPDAT5, LuPDAT6, and RcPDAT3 [6,29,30]. Interestingly, all these PDATs fall within the clade II in our tree (Figure 2). In addition to the possible nonfunctionalization of clade II PDATs, we speculate that these PDATs might have algal-like lipase activities due to their phylogenetic proximity to algal sequences (Figure 2), but more direct enzyme activity assays are needed to investigate this hypothesis. The PDAT members from the three *Avena* species clustered together, and phylogenetic analysis of oat, wheat, barley, all belonging to the Pooideae, share a closer evolutionary relationship (Figure 2). Additionally, our phylogeny are consistent with previous inference that the A and D subgenomes of hexaploid oat are more closely related to each other than to the C subgenome [31].

Although the involvement of PDAT in enhancing lipid production has been reported in several oil crops, the activities of AsPDATs in oat remained unclear. In this study, the expression levels of *AsPDAT* genes increased gradually and peaked during the late stage of seed development (Figure 3A,B), suggesting that *AsPDATs* may promote seed lipid accumulation in a developmentally dependent way, which was consistent with the transcript profiles in Arabidopsis [12], flax [29] and *Sapium sebiferum* [14]. Our investigation further suggested that the three *AsPDAT* homeologous genes may be functionally redundant due to their similar expression patterns (Figure 3) and high sequence similarities (96–98%). The minimal variation in physicochemical properties further indicated functional conservation and redundancy within AsPDAT proteins. Notably, the *AsPDAT-5C* gene constantly exhibited the highest expression patterns (Figure 3), suggesting that it may be the major PDAT isoform in oat. Heterologous expression in the mutant yeast H1246 and subcellular localization assay suggested that AsPDAT-5C contributes to ER-localized PDAT activity for TAG accumulation in oat (Figure 4). Previous research has indicated that neither mutation nor overexpression of *AtPDAT1* had overt impact on Arabidopsis seed lipid phenotype [6,7], but silencing of *AtPDAT1* led to a 63% reduction in total lipid content in the Arabidopsis *dgat1* mutant background [12], demonstrating that AtPDAT1 is a key determinant of seed lipid biosynthesis in the absence of DGAT1 activity. However, our data showed that ectopic expression of *AsPDAT-5C* in tobacco could give a 30% increase in seed lipid content (Figure 5B). This is consistent with the genetic manipulations of *PDAT* genes that are expected to elevate seed lipid biosynthesis, including overexpression of *CsaPDAT1* in *Camelina sativa* [20] and overexpression of *SsPDAT1* in *Brassica napus* [14]. In this study, *AsPDATs* genes exhibited the highest expression level in oat leaves not in seeds, reflecting their fundamental roles in association with lipid accumulation in vegetative organs (Figure 3A,B). Indeed, overexpression of *AsPDAT-5C* also significantly increased lipid levels in tobacco leaves (Figure 5E). Supporting this result, expression of *AtPDAT1* in Arabidopsis caused a large increase in its leaf lipid content [13]. Although lipid concentrations are usually low in vegetative organs, bioengineering efforts to bolster lipid production in leafy biomass crops are a promising sustainable source of renewable feedstocks [32]. From a biotechnological point of view, this clarification of the role of *AsPDAT-5C* may provide a desirable target for future genetic engineering to enhance lipid accumulation in both seed and leaf organs in biomass crops.

Although functional redundancy between DGAT and PDAT in lipid synthesis was proposed over two decades ago, the presence of varied expression indicates the crucial role of *PDAT* in the plant vegetative stages. Previously, metabolic and biochemical profiles have revealed enhanced lipid synthesis in plant vegetative organs under a variety of environmental stresses, confirming the role of lipid metabolism in stress tolerance improvement [33]. However, it is unknown whether PDAT-mediated lipid mechanism is a controlled process required to tolerate abiotic stresses. Through gene expression analysis, we determined that *AsPDAT* genes were significantly induced under multiple unfavorable conditions (Figure 3C–F). Enhanced cold tolerance has been previously proposed in *AtPDAT1* overexpression in Arabidopsis plants [34,35]. Similarly, our transgenic tobacco plants overexpressing *AsPDAT-5C* also performed better under cold conditions (Figure 6). When suffering from changes in temperatures, plants have evolved a series of sophisticated mechanisms to cope with cold stress, one adaptive strategy by which plants respond to low temperature change is to increase fatty acid unsaturation levels to maintain membrane integrity and fluidity, which is beneficial for improving photosynthetic efficiency [36]. Indeed, our studies found that unsaturated fatty acids were increased in the overexpression plants compared to that of wild type at low temperatures (Figure 6I). This observation is in accordance with both the function of PDAT as an enzyme having a substrate preference for unsaturated fatty acids and the function of the ER as the cellular site for membrane lipid biosynthesis [37]. In addition, cold stress could cause oxidative damage due to excess ROS accumulation. Our study revealed that overexpression plants exhibited enhanced peroxidase activities (POD, SOD and CAT) and lower levels of MDA and H_2_O_2_ (Figure 6). Thus, we proposed that *AsPDAT-5C*-mediated cold tolerance could also be partially explained by a stronger ROS scavenging capacity.

Previous observations strongly suggested that in addition to its common role in lipid synthesis, PDAT may contribute to adaptation to abiotic stresses in plants [3]. However, the role of PDAT in nutrient deficiency is poorly understood. In accordance with the altered expression pattern (Figure 3F), our data revealed a novel positive effect of *AsPDAT-5C* on low-P stress (Figure 7). Compared to the lipid composition in WT leaves, overexpression of *AsPDAT-5C* facilitated the degradation of phospholipids during P deficiency (Figure 7G), which likely resulted from its phospholipase activity using phospholipids as substrates. Phospholipids constitute about 1/3 of organic P in plant organs, and degradation of phospholipids to release P for other critical cellular processes is an effective strategy to cope with P-limited environments in plants [37,38]. As expected, the elevated accumulation of non-P neutral lipids was due to the acyltransferase activity of AsPDAT-5C. As a major class of neutral lipids, the importance of TAG metabolism in P stress response has begun to be appreciated [39,40], but the specific enzymes and underlying mechanisms have not been investigated. The present study provides evidence that AsPDAT-5C mediates TAG biosynthesis, but it also displays actions on phospholipid hydrolysis; thus, we cannot rule out the possibility that other genes for TAG production, like *DGAT*, help plants adapt to low-P environments. Glycolipids, another important class of non-P-containing lipids, are mainly utilized to functionally compensate for the lack of phospholipids and thus hold prime importance under P deficiency [41]. As reflected by the upregulation of glycolipids under low-P conditions (Figure 7H), our results suggest that oat *AsPDAT-5C* may cooperate with other genes involved in glycolipid biosynthesis to maintain the balance of lipid metabolism and enhance P utilization efficiency in plants under low-P conditions. This assumption is supported by the observation that tobacco galactolipids (MGDGs) biosynthetic gene, *NtMGD2*, a P-deficiency-responsive gene, showed induced expression in *AsPDAT-5C* OE plants. Therefore, the enhanced stress tolerance in *AsPDAT-5C* overexpression lines may not be solely attributable to this single gene but could result from its ability to orchestrate or synergize with a broader transcriptional and metabolic reprogramming involving these other critical genes.

## 4. Materials and Methods

### 4.1. Sequence Acquisition and Bioinformatics Analysis of PDAT Family Genes

Genomic files of oat (*A. sativa*), *A. longiglumis*, and *A. insularis* were downloaded from the OatOmics (http://www.oatomics.com, accessed on 10 August 2024). The genomic sequences of other species were downloaded from the public database Phytozome (https://phytozome-next.jgi.doe.gov/, accessed on 10 August 2024). The amino acid sequences of Arabidopsis AtPDAT proteins retrieved from TAIR (https://www.arabidopsis.org/, accessed on 10 August 2024) were used as queries to identify candidate PDAT orthologs in all listed plant species (Supplementary Table S1) by the BLAST approach. The presence of a transmembrane region and conserved LCAT signature in all candidate sequences was determined using InterPro (https://www.ebi.ac.uk/interpro/, accessed on 10 August 2024) and SMART database (http://smart.embl-heidelberg.de/, accessed on 10 August 2024). Multiple sequence alignments were performed using the MAFFT versions 7.526. The sequence identity was determined using EMBOSS Needle (https://www.ebi.ac.uk/jdispatcher/psa/emboss_needle, accessed on 10 August 2024). The phylogenetic tree was constructed using the Maximum Likelihood (ML) method with the Jones-Taylor-Thornton model, nearest-neighbour interchange, uniform rates and 1000 bootstrap replicates in MEGA11.0 software [42]. Visualization of the tree was performed using iTOL (https://itol.embl.de/, accessed on 10 August 2024). The physical and chemical properties of AsPDAT proteins in oat, including molecular weight, instability index, hydrophobicity, and isoelectric point, were predicted by using the ExPASy server (https://www.expasy.org/, accessed on 10 August 2024). Gene structure annotation files of *AsPDAT* genes retrieved from OatOmics were used to visualize the diagram of exon/intron distribution in Gene Structure Display Server (http://gsds.cbi.pku.edu.cn/, accessed on 10 August 2024). Subcellular localization was predicted using the Cell-PLoc 2.0 package (http://www.csbio.sjtu.edu.cn/bioinf/Cell-PLoc-2/, accessed on 10 August 2024).

### 4.2. Plant Materials and Growth Conditions

All experiments were carried out in the spring of 2023 at the experimental station of Shanxi Agricultural University, Shanxi, China. The seeds of oat cultivar CEav5651 were obtained from the agricultural genetic resources center, Shanxi Agricultural University. Oat seeds were planted in a 3:1 (*w*/*w*) mixture of soil and sand and placed in a greenhouse with 16 h light/8 h dark and 80% relative humidity at 22 °C. The photoperiod of 16 h per day was maintained under natural light supplemented with white fluorescent tube at an approximate photon flux intensity of 220 μmol·m^−2^·s^−1^. To perform organ-specific expression profiling of *AsPDAT* genes, different oat organs were collected from roots, stems, leaves, and spikes at the heading stage. The seed samples were collected at three development stages (10, 20, and 30 days after flowering, DAF). These samples were immediately snap-frozen in liquid nitrogen and stored at −80 °C for future use. *Nicotiana benthamiana* and *Nicotiana tabacum* employed in this study were grown in a manual climatic box (RDN-560C-4, Ningbo, China) under a photoperiod of 16 h light and 8 h darkness, photon flux intensity of 250 µmol∙m^−2^∙s^−1^, constant temperature of 25 °C, and relative humidity of 70%. A hydroponic cultivation experiment was performed to conduct stress treatment in oat. Oat seeds were firstly germinated on filter paper soaked with distilled water and incubated in the dark for 96 h at 24 °C. Later, the germinated seeds were transplanted into hydroponic buckets containing Half-strength Hoagland’s modified nutrient solution (pH 6.0) and placed in a greenhouse with 16 h light/8 h dark and 80% relative humidity at 22 °C. The photoperiod of 16 h per day was maintained under natural light supplemented with white fluorescent tube at an approximate photon flux intensity of 220 μmol·m^−2^·s^−1^. Half-strength Hoagland’s modified nutrient solution contains 2.5 mM KNO_3_, 0.5 mM NH_4_NO_3_, 0.5 mM KH_2_PO_4_, 1 mM MgSO_4_, 0.15 mM FeEDTA, 5 μM KI, 0.1 mM H_3_BO_3_, 0.15 mM MnSO_4_, 0.05 mM ZnSO_4_, 0.1 μM CoCl_2_, 20 mM Na_2_MoO_4_, 0.01 μM CuSO_4_, and 3 mM Ca(NO_3_)_2_. When oat seedlings had grown for four weeks, diverse stress treatments were initiated, including salt stress (200 mM NaCl), drought (18% PEG6000, *w*/*v*, −0.75 MPa), and cold (4 °C). Additionally, for phosphorus (P) deficiency treatment, oat seedlings were transferred to a P-deficient solution, which was identical to the half-strength Hoagland’s modified solution except that 0.5 mM KH_2_PO_4_ was replaced by 0.5 mM KCl. For nitrogen (N) deprivation treatment, oat seedlings were transferred to N-deficient solution where KNO_3_ (2.5 mM), NH_4_NO_3_ (0.5 mM), and Ca(NO_3_)_2_ (3 mM) in the half-strength Hoagland’s modified nutrient medium were replaced with 2.5 mM KCl, 0.5 mM NH_4_Cl, and 3 mM CaCl_2_, respectively. For wild-type and transgenic tobacco (*N. benthamiana*) lines, plants were initially grown in vermiculite and irrigated weekly with half-strength Hoagland’s modified nutrient solution. The 40-day-old tobacco seedlings were prepared and treated at 4 °C for cold stress, with normal growth conditions as the control. Leaf samples were collected at 72 h for further use. The treatment conditions of P deprivation and N deprivation were irrigated with P-deficient half-strength Hoagland’s modified nutrient solution, with normal growth conditions as the control. Leaf samples were collected at 15 day for further use. Unless otherwise specified, all general chemicals and reagents were obtained from Beijing Solarbio Science & Technology (Solarbio, Beijing, China).

### 4.3. RNA Isolation and Gene Expression Analysis

Total RNA of the above-mentioned samples was isolated using the RNA Extraction Kit (TIANGEN, Beijing, China). The Prime Script RT Reagent Kit (Takara Bio, Dalian, China) was used to synthesize cDNA and SYBR Premix Ex Taq II (Takara Bio, Dalian, China) was used for quantitative real-time PCR (qRT-PCR) analysis on a CFX96 Real-Time PCR Detection System (BIO-RAD, Hercules, CA, USA). The *AsActin* gene (GenBank accession number: KP257585.1) was chosen as an internal control and the 2^−ΔΔCT^ method was used to quantify the relative expression of *AsPDAT* genes in oat. *NtTubulin* (GenBank accession number: N181029A17) was used as an internal control to investigate gene expression in transgenic tobacco plants overexpressing *AsPDAT-5C* and WT plants. Each sample was assayed with three biological replicates. Primers used were shown in Supplementary Table S2. The transcriptome data used in this study were obtained from Data Center’s Genome Sequence Archive (http://bigd.big.ac.cn/, accessed on 25 November 2025) under accession numbers SAMN19582573 and SAMN19582574. Raw transcriptome data were first subjected to quality control using FastQC v0.12.1 (https://www.bioinformatics.babraham.ac.uk/projects/fastqc/, accessed on 25 November 2025). Subsequently, the clean reads were aligned to the reference genome with HISAT2 v2.2.1 (https://daehwankimlab.github.io/hisat2/, accessed on 25 November 2025). Read counts for each gene were generated with HTSeq-count, and the final gene expression levels were calculated as fragments per kilobase of transcript per million mapped reads (FPKM) values using R (https://www.r-project.org/, accessed on 25 November 2025). This established workflow ensures the accuracy and reproducibility of the results.

### 4.4. Subcellular Localization and AsPDAT-5C Expression in Yeast Mutant H1246

To determine the subcellular localization of AsPDAT-5C, its coding sequence (CDS) was amplified and cloned into the plant expression vector pCAMBIA1300-35S-GFP, generating a C-terminal fusion construct designated pCAMBIA1300-35S::*AsPDAT-5C*-GFP. For precise organelle localization, the endoplasmic reticulum (ER) marker construct pCAMBIA1300-35S::ER-mCherry-HDEL (Coolaber Biological Technology, Beijing, China) was used. Each construct was then individually transformed into *Agrobacterium tumefaciens* strain GV3101 via the freeze-thaw method to generate two distinct bacterial strains. For infiltration, Agrobacterium suspensions harboring the respective constructs were mixed in equal volumes (based on OD600) and co-infiltrated into the leaves from four-week-old *Nicotiana benthamiana* plants. After 48 h of incubation, epidermal cells were imaged using a confocal laser-scanning microscope (Leica TCS SP8, Leica Microsystems, Wetzlar, Germany). The co-localization of the *AsPDAT-5C*-GFP fusion signal with the ER-mCherry marker was analyzed to confirm ER localization.

For functional complementation assays, the CDS of *AsPDAT-5C* was subcloned into the galactose-inducible yeast expression vector pYES2.0, yielding the construct pYES2.0-*AsPDAT-5C*. This construct was transformed into the TAG-deficient *Saccharomyces cerevisiae* H1246 quadruple mutant using the polyethylene glycol/lithium acetate method [43]. Tragansformants were selected on synthetic complete (SC) medium lacking uracil (SC-Ura). For induction, single colonies were inoculated into liquid SC-Ura medium containing 2% (*w*/*v*) galactose and cultured at 30 °C with shaking at 180 rpm for 72 h in the dark using a shaker incubator (YIHENG Technology, Shanghai, China). To visualize neutral lipid accumulation, induced yeast cells were stained with the lipid droplet-specific fluorescent dye BODIPY 505/515 and observed under the confocal microscope (Leica TCS SP8, Leica Microsystems, Wetzlar, Germany). Total TAG was extracted from the yeast cells and quantified using a thin-layer chromatography (TLC)-based densitometry method, as previously described [43]. All experiments were performed with three biological replicates. Primers used for vector construction are listed in Supplementary Table S2.

### 4.5. Vector Construction and Transgenic Plant Generation

The amplified *AsPDAT-5C* coding sequence was cloned into the pCAMBIA1303 vector driven by the 35S promoter for constitutive overexpression. The final construct (pCAMBIA1303-*AsPDAT-5C*) was introduced into tobacco (*N. tobaccum*) WT for stable genetic transformation using the Agrobacterium (GV3101)-mediated method as described previously [44]. The *AsPDAT-5C* overexpressed tobacco plants were obtained and characterized by reverse transcription PCR (RT-PCR). Two independent T2 transgenic lines showing higher expression levels of *AsPDAT-5C* were used in the experiments. Wild type (WT) tobacco plants were used as control. The primer sequences are listed in Supplementary Table S2.

### 4.6. Total Lipid, Soluble Sugar and Protein Measurement

Total lipid content was determined following a previously established protocol [44]. Briefly, freeze-dried mature tobacco leaf and seed samples harvested from WT and *AsPDAT-5C*-overexpressing lines were ground into powder. Then, 0.05 g powder was weighed and dissolved in a 7.5 mL of methanol/chloroform (2:1, *v*/*v*) mixture. After overnight incubation at 37 °C, the mixture was centrifuged at 5000 rpm for 8 min. The supernatant was added with 5 mL of chloroform and 9 mL of a 1% NaCl (*w*/*v*). After centrifugation at 8000 rpm for 10 min, the extract was separated into two distinct phases: an upper aqueous phase and a lower organic phase containing the total lipid. The upper aqueous phase was discarded. The lower lipid-containing organic phase was transferred into a pre-weighed glass vial (weight recorded as W1). The solvent was completely evaporated under a gentle stream of nitrogen gas. The vial with the extracted lipid was then reweighed (W2). Total lipid content was determined using the following formula: total lipid content (%) = [(W2 − W1)/0.05] × 100. Following the instructions of the reagent kit, soluble sugar and protein contents from freeze-dried mature tobacco leaf and seed samples were determined using the Plant Soluble Sugar Content Assay Kit (Solarbio, Beijing, China) and Bradford Protein Assay Kit (GenStar, Beijing, China), respectively. All subsequent content calculations are expressed on a dry weight basis.

### 4.7. Fatty Acid Profile and Lipid Composition Analysis

Preparation of fatty acid methyl esters (FAMEs) was carried out using a previously established protocol [43]. Heptadecanoic acid (C17:0) was used as an internal standard during lipid extraction. The fatty acid profiles of each sample were analyzed on an Agilent 7890B gas chromatograph (GC) equipped with a 5975B mass selective detector and a G3903-63011 capillary column (Agilent Technologies, Santa Clara, CA, USA). Relative quantification of individual fatty acids in samples was achieved by normalizing the retention time and peak area of the internal standard (C17:0). Lipid component separation was achieved using the Cleanert Silica solid phase extraction (SPE) Cartridge (Agela, Tianjin, China), similar to the method described previously [39]. Briefly, the neutral lipids, glycolipids, and phospholipids were separated by stepwise gradient elution from chloroform, acetone, and methanol, respectively. The different lipid components were quantified by weighing after evaporation under nitrogen.

### 4.8. Analysis of Oxidative Stress Parameters

The malondialdehyde (MDA) content was quantified using the thiobarbituric acid (TBA) method as described by Heath and Packer [45]. Briefly, 0.1 g fresh leaf samples was homogenized in an extraction solution containing 10% (*w*/*v*) trichloroacetic acid (TCA). The homogenate was then mixed with 5% (*w*/*v*) TBA solution and incubated in a boiling water bath for 30 min. After cooling, the mixture was centrifuged at 12,000× *g* for 30 min. The absorbance of the supernatant was measured at 450 nm, 532 nm, and 600 nm for the calculation of MDA content. The hydrogen peroxide (H_2_O_2_) content was determined spectrophotometrically following the protocol of Velikova et al. [46]. Briefly, 0.1 g fresh leaf samples were homogenized in ice-cold 0.1% (*w*/*v*) trichloroacetic acid and centrifuged at 12,000× *g* for 20 min at 4 °C. The supernatant was incubated with a reaction buffer consisting of 10 mM potassium phosphate buffer (pH 7.0) and 1 mM potassium iodide (KI). The absorbance of the reaction mixture at 390 nm was measured to determine H_2_O_2_ content. The enzyme activities of superoxide dismutase (SOD, EC 1.15.1.1, Cat: A001-3-2), peroxidase (POD, EC 1.11.1.7, Cat: A084-3-1), and catalase (CAT, EC 1.11.1.6, Cat: A007-1-1) in tobacco seedling leaves were measured using the corresponding assay kit (Nanjing Jiancheng Bioengineering Institute, Nanjing, China) according to the manufacturer’s instructions. For the ion leakage assay, leaves of tobacco seedlings were collected in 15 mL tubes containing 10 mL of ddH_2_O. The initial conductivity of the solution was measured and designated as S0. The tubes were then shaken at room temperature for 30 min, after which the conductivity was measured and recorded as S1. Subsequently, the samples were boiled for 30 min, cooled to room temperature with shaking, and the final conductivity (S2) was measured. Ion leakage was calculated according to the formula Ion leakage (%) = [(S1 − S0)/(S2 − S0)] × 100.

## 5. Conclusions

This study presented the first genome-wide identification of the PDAT gene family in hexaploid cultivated oat, a cereal renowned for its high seed lipid content. We systematically identified three homologous *AsPDAT* genes derived from polyploidization, which were distributed across subgenomes and exhibited high conservation in sequence and structure. Phylogenetic and expression profiling revealed that these homologs likely retain similar functions, with *AsPDAT-5C* showing consistently higher transcript levels under both developmental and abiotic stress conditions. Functional characterization confirmed that *AsPDAT-5C* encodes an endoplasmic reticulum-localized enzyme with authentic PDAT activity, capable of complementing TAG biosynthesis in yeast. Crucially, stable overexpression of *AsPDAT-5C* in tobacco significantly enhanced lipid accumulation in both leaves and seeds and substantially improved plant tolerance to cold and P deficiency stresses. Mechanistic investigations indicated that this multi-stress resilience is mediated through *AsPDAT-5C*-driven lipid remodeling, including increased fatty acid unsaturation for membrane integrity under cold and a shift from phospholipids to non-phosphorus lipids for phosphate homeostasis under P limitation. Our results provide foundational insights into the AsPDAT gene family and establish *AsPDAT-5C* as a key regulator integrating lipid metabolism with environmental adaptation. This work not only offers a valuable genetic resource for understanding lipid dynamics in polyploid cereals but also highlights *AsPDAT-5C* as a promising biotechnological target for the dual improvement of lipid yield and abiotic stress resilience in crops.

## Figures and Tables

**Table 1:** Genomic information and protein characteristics of oat *AsPDAT* genes.

Gene ID (OatOmics)	Gene Name	Chromosome	Amino Acid Number	Molecular Weight (Da)	pI	Instability Index	Aliphatic Index	Grand Average of Hydropathicity	Predictions for Subcellular Localization
1059_5A004970	AsPDAT-5A	5A	694	76,474.89	5.8	44.04	73.89	−0.323	ER
1059_5C004228	AsPDAT-5C	5C	690	76,019.45	5.91	42.93	73.9	−0.325	ER
1059_5D004221	AsPDAT-5D	5D	699	76,875.18	5.8	43	73.22	−0.324	ER

Note: ER indicates endoplasmic reticulum.

## Data Availability

The original contributions presented in this study are included in the article/Supplementary Material. Further inquiries can be directed to the corresponding authors.

## Supplementary Materials

The following supporting information can be downloaded at https://www.mdpi.com/article/10.3390/plants15010035/s1: Figure S1: Molecular identification of transgenic tobacco plants. (A): Detection of *AsPDAT-5C* transgenic tobacco lines at the genomic level. (B): Detection of *AsPDAT-5C* transgenic tobacco lines at the transcriptional level. Total RNA and genomic DNA were extracted from mature seeds of T2 generation plants. Lane M: DNA size marker. Lane 1–5: *AsPDAT-5C* transgenic plants. Lane 6: pCAMBIA1303 + *AsPDAT-5C* recombinant plasmid (positive control). Lane 7: Wild-type tobacco (negative control). (C): Relative expression levels of *AsPDAT-5C* gene. Data are means ± SD (n = 3); the statistical comparisons were performed using Student’s *t*-test (** *p* < 0.01); Table S1: Species and data information for PDAT sequences used in this study; Table S2: Primers used in this study.

## Abbreviations

The following abbreviations are used in this manuscript:

PDAT	Phospholipid:Diacylglycerol Acyltransferase
DGAT	Diacylglycerol Acyltransferase
TAG	Triacylglycerol
LCAT	Lecithin:cholesterol Acyltransferase
TMD	Transmembrane Domain
DAF	Days After Flowering
ER	Endoplasmic Reticulum

## References

[1] Yang Y., Benning C. (2018). Functions of triacylglycerols during plant development and stress. Curr. Opin. Biotechnol..

[2] Xu Y., Caldo K.M.P., Pal-Nath D., Ozga J., Lemieux M.J., Weselake R.J., Chen G. (2018). Properties and Biotechnological Applications of Acyl-CoA:diacylglycerol Acyltransferase and Phospholipid:diacylglycerol Acyltransferase from Terrestrial Plants and Microalgae. Lipids.

[3] Sah S.K., Fan J., Blanford J., Shanklin J., Xu C. (2024). Physiological Functions of Phospholipid:Diacylglycerol Acyltransferases. Plant Cell Physiol..

[4] Pan X., Peng F.Y., Weselake R.J. (2015). Genome-wide analysis of phospholipid: Diacylglycerol acyltransferase (PDAT) genes in plants reveals the eudicot-wide PDAT gene expansion and altered selective pressures acting on the core eudicot PDAT paralogs. Plant Physiol..

[5] Dahlqvist A., Stahl U., Lenman M., Banas A., Lee M., Sandager L., Ronne H., Stymne S. (2000). Phospholipid:diacylglycerol acyltransferase: An enzyme that catalyzes the acyl-CoA-independent formation of triacylglycerol in yeast and plants. Proc. Natl. Acad. Sci. USA.

[6] Stahl U., Carlsson A.S., Lenman M., Dahlqvist A., Huang B., Banas W., Banas A., Stymne S. (2004). Cloning and functional characterization of a phospholipid:diacylglycerol acyltransferase from Arabidopsis. Plant Physiol..

[7] Mhaske V., Beldjilali K., Ohlrogge J., Pollard M. (2005). Isolation and characterization of an Arabidopsis thaliana knockout line for phospholipid: Diacylglycerol transacylase gene (At5g13640). Plant Physiol. Biochem..

[8] Li R., Yu K., Hildebrand D.F. (2010). DGAT1, DGAT2 and PDAT expression in seeds and other organs of epoxy and hydroxy fatty acid accumulating plants. Lipids.

[9] van Erp H., Bates P.D., Burgal J., Shockey J., Browse J. (2011). Castor phospholipid:diacylglycerol acyltransferase facilitates efficient metabolism of hydroxy fatty acids in transgenic Arabidopsis. Plant Physiol..

[10] Yoon K., Han D., Li Y., Sommerfeld M., Hu Q. (2012). Phospholipid:diacylglycerol acyltransferase is a multifunctional enzyme involved in membrane lipid turnover and degradation while synthesizing triacylglycerol in the unicellular green microalga Chlamydomonas reinhardtii. Plant Cell.

[11] Oelkers P., Cromley D., Padamsee M., Billheimer J.T., Sturley S.L. (2002). The DGA1 gene determines a second triglyceride synthetic pathway in yeast. J. Biol. Chem..

[12] Zhang M., Fan J., Taylor D.C., Ohlrogge J.B. (2009). DGAT1 and PDAT1 acyltransferases have overlapping functions in Arabidopsis triacylglycerol biosynthesis and are essential for normal pollen and seed development. Plant Cell.

[13] Fan J., Yan C., Zhang X., Xu C. (2013). Dual role for phospholipid:diacylglycerol acyltransferase: Enhancing fatty acid synthesis and diverting fatty acids from membrane lipids to triacylglycerol in Arabidopsis leaves. Plant Cell.

[14] Zhou B., Fei W., Yang S., Yang F., Qu G., Tang W., Ou J., Peng D. (2020). Alteration of the fatty acid composition of *Brassica napus* L. via overexpression of phospholipid: Diacylglycerol acyltransferase 1 from *Sapium sebiferum* (L.) Roxb. Plant Sci..

[15] Fenyk S., Woodfield H.K., Romsdahl T.B., Wallington E.J., Bates R.E., Fell D.A., Chapman K.D., Fawcett T., Harwood J.L. (2022). Overexpression of phospholipid: Diacylglycerol acyltransferase in Brassica napus results in changes in lipid metabolism and oil accumulation. Biochem. J..

[16] Wang J., Ren H., Shi Z., Phillip F.O., Liu S., Zhang W., Wang X., Bao X., Guo J. (2024). Exploring Functional Gene XsPDAT1’s Involvement in Xanthoceras sorbifolium Oil Synthesis and Its Acclimation to Cold Stress. Forests.

[17] Boyle N.R., Page M.D., Liu B., Blaby I.K., Casero D., Kropat J., Cokus S.J., Hong-Hermesdorf A., Shaw J., Karpowicz S.J. (2012). Three acyltransferases and nitrogen-responsive regulator are implicated in nitrogen starvation-induced triacylglycerol accumulation in Chlamydomonas. J. Biol. Chem..

[18] Mueller S.P., Unger M., Guender L., Fekete A., Mueller M.J. (2017). Phospholipid:Diacylglycerol Acyltransferase-Mediated Triacylglyerol Synthesis Augments Basal Thermotolerance. Plant Physiol..

[19] Demski K., Losiewska A., Jasieniecka-Gazarkiewicz K., Klinska S., Banas A. (2020). Phospholipid:Diacylglycerol Acyltransferase1 Overexpression Delays Senescence and Enhances Post-heat and Cold Exposure Fitness. Front. Plant Sci..

[20] Yuan L., Mao X., Zhao K., Ji X., Ji C., Xue J., Li R. (2017). Characterisation of phospholipid: Diacylglycerol acyltransferases (PDATs) from Camelina sativa and their roles in stress responses. Biol. Open.

[21] Hernandez M.L., Moretti S., Sicardo M.D., Garcia U., Perez A., Sebastiani L., Martinez-Rivas J.M. (2021). Distinct Physiological Roles of Three Phospholipid:Diacylglycerol Acyltransferase Genes in Olive Fruit with Respect to Oil Accumulation and the Response to Abiotic Stress. Front. Plant Sci..

[22] Peng J., Lei X., Liu T., Xiong Y., Wu J., Xiong Y., You M., Zhao J., Zhang J., Ma X. (2025). Integration of machine learning and genome-wide association study to explore the genomic prediction accuracy of agronomic trait in oats (*Avena sativa* L.). Plant Genome.

[23] Zhou Z., Kaur R., Donoso T., Ohm J.B., Gupta R., Lefsrud M., Singh J. (2024). Metabolic engineering-induced transcriptome reprogramming of lipid biosynthesis enhances oil composition in oat. Plant Biotechnol. J..

[24] Yang Z., Liu X., Li N., Du C., Wang K., Zhao C., Wang Z., Hu Y., Zhang M. (2019). WRINKLED1 homologs highly and functionally express in oil-rich endosperms of oat and castor. Plant Sci..

[25] Hu H., Gutierrez-Gonzalez J.J., Liu X., Yeats T.H., Garvin D.F., Hoekenga O.A., Sorrells M.E., Gore M.A., Jannink J.L. (2020). Heritable temporal gene expression patterns correlate with metabolomic seed content in developing hexaploid oat seed. Plant Biotechnol. J..

[26] Hayden D.M., Rolletschek H., Borisjuk L., Corwin J., Kliebenstein D.J., Grimberg A., Stymne S., Dehesh K. (2011). Cofactome analyses reveal enhanced flux of carbon into oil for potential biofuel production. Plant J..

[27] Vanhercke T., Dyer J.M., Mullen R.T., Kilaru A., Rahman M.M., Petrie J.R., Green A.G., Yurchenko O., Singh S.P. (2019). Metabolic engineering for enhanced oil in biomass. Prog. Lipid Res..

[28] Falarz L.J., Xu Y., Caldo K.M.P., Garroway C.J., Singer S.D., Chen G. (2020). Characterization of the diversification of phospholipid:diacylglycerol acyltransferases in the green lineage. Plant J..

[29] Pan X., Siloto R.M., Wickramarathna A.D., Mietkiewska E., Weselake R.J. (2013). Identification of a pair of phospholipid:diacylglycerol acyltransferases from developing flax (*Linum usitatissimum* L.) seed catalyzing the selective production of trilinolenin. J. Biol. Chem..

[30] Kim H.U., Lee K.R., Go Y.S., Jung J.H., Suh M.C., Kim J.B. (2011). Endoplasmic reticulum-located PDAT1-2 from castor bean enhances hydroxy fatty acid accumulation in transgenic plants. Plant Cell Physiol..

[31] Peng Y., Yan H., Guo L., Deng C., Wang C., Wang Y., Kang L., Zhou P., Yu K., Dong X. (2022). Reference genome assemblies reveal the origin and evolution of allohexaploid oat. Nat. Genet..

[32] Singh R., Arora A., Singh V. (2021). Biodiesel from oil produced in vegetative organs of biomass—A review. Bioresour. Technol..

[33] Nam J.W., Lee H.G., Do H., Kim H.U., Seo P.J. (2022). Transcriptional regulation of triacylglycerol accumulation in plants under environmental stress conditions. J. Exp. Bot..

[34] Klinska-Bachor S., Kedzierska S., Demski K., Banas A. (2023). Phospholipid:diacylglycerol acyltransferase1-overexpression stimulates lipid turnover, oil production and fitness in cold-grown plants. BMC Plant Biol..

[35] Shomo Z.D., Mahboub S., Vanviratikul H., McCormick M., Tulyananda T., Roston R.L., Warakanont J. (2024). All members of the Arabidopsis DGAT and PDAT acyltransferase families operate during high and low temperatures. Plant Physiol..

[36] Zhang L., Wang S., Bai B., Chen Y., Xiang Z., Chen C., Kuang X., Yang Y., Fu J., Chen L. (2024). OsKASI-2 is required for the regulation of unsaturation levels of membrane lipids and chilling tolerance in rice. Plant Biotechnol. J..

[37] Ghosal A., Banas A., Ståhl U., Dahlqvist A., Lindqvist Y., Stymne S. (2007). Saccharomyces cerevisiae phospholipid:diacylglycerol acyl transferase (PDAT) devoid of its membrane anchor region is a soluble and active enzyme retaining its substrate specificities. Biochim. Biophys. Acta.

[38] Liu C., Tai Y., Luo J., Wu Y., Zhao X., Dong R., Ding X., Zhao S., Luo L., Liu P. (2022). Integrated multi-omics analysis provides insights into genome evolution and phosphorus deficiency adaptation in pigeonpea (*Cajanus cajan*). Hortic. Res..

[39] Wen J., Chai X., Huang X., Yang H., Lei T., Dong S., Li R., Wang J., Zhou Y. (2025). PfPAH1-1 gene enhances plant tolerance to low phosphate stress by modulating cell membrane lipid remodeling. Plant Physiol. Biochem..

[40] Pant B.D., Burgos A., Pant P., Cuadros-Inostroza A., Willmitzer L., Scheible W.R. (2015). The transcription factor PHR1 regulates lipid remodeling and triacylglycerol accumulation in Arabidopsis thaliana during phosphorus starvation. J. Exp. Bot..

[41] Kobayashi K., Awai K., Nakamura M., Nagatani A., Masuda T., Ohta H. (2009). Type-B monogalactosyldiacylglycerol synthases are involved in phosphate starvation-induced lipid remodeling, and are crucial for low-phosphate adaptation. Plant J..

[42] Tamura K., Stecher G., Kumar S. (2021). MEGA11: Molecular evolutionary geneticsanalysis version 11. Mol. Biol. Evol..

[43] Zhou Y., Huang X., Sun Y., Chai X., Wen J., Yang Z., Xue J., Zhang X., Jia X., Wang J. (2025). Three Distinctive Diacylglycerol Acyltransferases (DGAT1, DGAT2, and DGAT3) from Perilla frutescens and Their Potential in Metabolic Engineering for Designed Oil Production. J. Agric. Food Chem..

[44] Wang Z.L., Su Y.T., Wang L.L., Ma L., Sun Y., Li R.Z., Ge L.P. (2024). Characterization of GPAT gene family in *Euphorbia lathyris* L. and elucidating the role of ElGPAT9 in the biosynthesis of oils and pollen viability. Ind. Crops Prod..

[45] Heath R.L., Packer L. (1968). Photoperoxidation in isolated chloroplasts. I. Kinetics and stoichiometry of fatty acid peroxidation. Arch. Biochem. Biophys..

[46] Velikova V., Yordanov I., Edreva A. (2000). Oxidative stress and some antioxidant systems in acid rain-treated bean plants. Plant Sci..

