# Supplementary figures and images for: Characterization of PDAT Genes in Oat (Avena sativa L.) and the Role of AsPDAT-5C in Lipid Biosynthesis and Abiotic Stress Response
Source: Plants (Basel). 2025 Dec 22;15(1):35. doi: 10.3390/plants15010035 (PMC12787530; doi:10.3390/plants15010035)

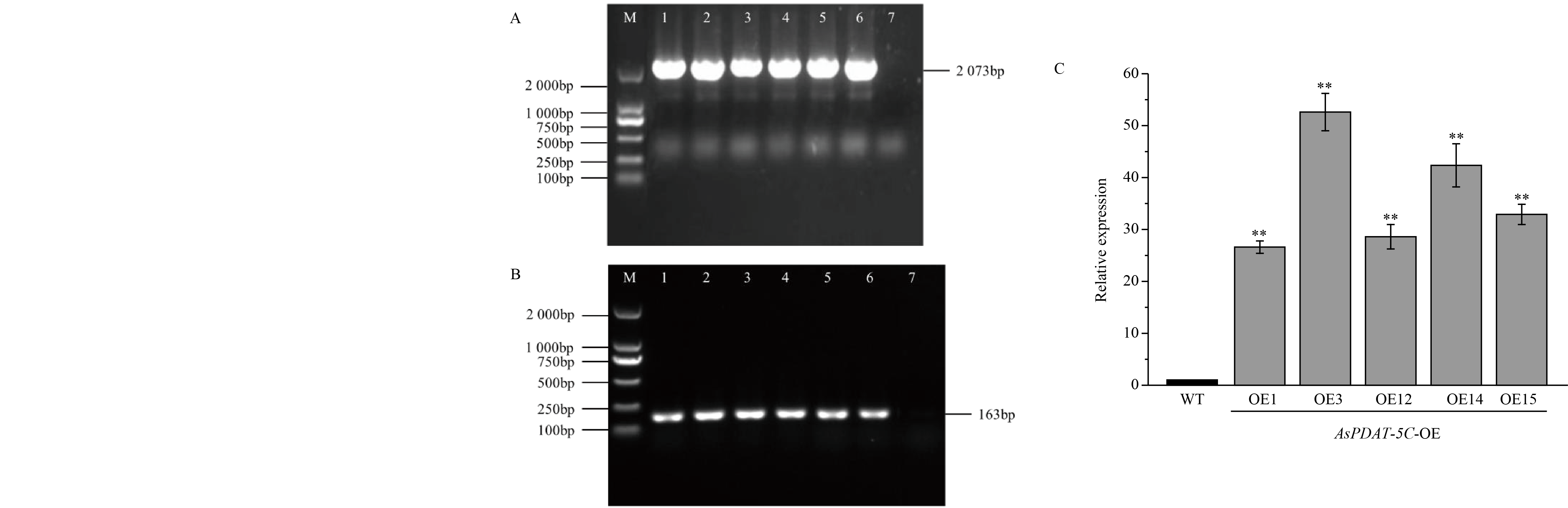

Supplement: Supplementary file 1 [file plants-15-00035-s001.zip › Supplementary Figure S1.tif]
